# Structural Properties Before and After Ripening of Ice Cream Made with Different Dairy Fat Bases

**DOI:** 10.3390/foods14183276

**Published:** 2025-09-22

**Authors:** Paulo Henrique da Silva Santos, Cristina Kaori Suzuki, Suzana Caetano da Silva Lannes

**Affiliations:** School of Pharmaceutical Sciences, University of Sao Paulo, São Paulo 05508-000, SP, Brazil; paulo_santos@usp.br (P.H.d.S.S.); cristinasuzuki@usp.br (C.K.S.)

**Keywords:** ice cream, dairy fat, fat crystallization, rheology, food structure

## Abstract

Ice cream is a frozen aerated dessert composed of milk solids, sugars, stabilizers, and fat—with the latter being a key component in defining its structural and sensory properties. This study evaluated the influence of four fat sources—low-trans vegetable fat (T1), butter (T2), UHT cream (T3), and fresh cream (T4)—on the physical and structural characteristics of ice cream, including overrun, melting resistance, texture, color, and rheology, at different stages of processing (before and after maturation). Oscillatory rheological analysis revealed predominantly elastic behavior (G′ > G″) after maturation in all samples, indicating a stable viscoelastic solid structure. Formulations containing T3 and T1 showed the highest overrun values, indicating greater air incorporation, whereas the butter-based formulation (T2) showed the lowest overrun values. Melting resistance followed the following order: T3 > T4 > T2 > T1; therein, the UHT cream formulation exhibited the greatest thermal stability, which was likely due to protein denaturation and aggregation induced by high-temperature processing. Texture analysis showed that the T1 formulation required the lowest maximum extrusion force, while T2 required the highest, reflecting an inverse correlation with overrun values. T1 also displayed the most distinct rheological profile, which was likely due to its specific crystallization behavior and reduced destabilization of the fat globule membrane—which favored the development of a more structured internal network. These findings demonstrate that both the source and processing of fat have a significant impact on the formation of the structural matrix and the final functional properties of ice cream. The results offer technical insights for the development of formulations tailored to specific physical characteristics, optimizing texture, stability, and performance throughout the production process.

## 1. Introduction

Ice cream is a widely consumed frozen food product that may or may not contain milk fat in its formulation. It is the most popular item among frozen dairy desserts, being available in various versions such as conventional ice cream—defined by minimum levels of fat and milk solids, or both, according to the regulations in the country of manufacture—as well as variations in high-fat content, low-fat content, no added sugars, or reduced sugar content, where it is offered in a wide range of flavors and formats [1,2]. The quality of ice cream is intrinsically associated with its thermal stability during storage, since temperature fluctuations can induce the growth or recrystallization of ice crystals, impairing texture and compromising the sensory experience of the consumer [1,3].

From a food processing perspective, ice cream is a highly complex system derived from a pasteurized colloidal emulsion. During freezing, carried out under continuous agitation and air incorporation, the product acquires desirable physical and sensory characteristics, resulting from the interaction between ice crystals, partially coalesced fat globules, a protein matrix, and air bubbles [2,4]. The microstructure of ice cream is strongly determined by fat crystals, which decisively influence its rheological and sensory properties. During cooling, fat crystallizes, and the formed nuclei initiate crystal growth, affecting interfacial properties and the final stability of the emulsion [1]. In this context, the use of emulsifiers and stabilizers is essential to prevent destabilization of the lipid phase, controlling interfacial tension and providing sufficient kinetic retention to maintain product stability [5,6].

The different fatty acids present in milk—short-, medium-, and long-chain—play distinct roles in ice cream characteristics, influencing everything from fat plasticity to sensory perception [7]. Buttermilk, in turn, due to its high polar lipid content, exhibits emulsifying and stabilizing properties, contributing to improved air incorporation and retention as well as emulsion stability [8,9].

Cream is a fat-rich dairy product obtained by separating the lipid phase of milk through appropriate technological methods, and it can be pasteurized or sterilized, as in Ultra-High-Temperature (UHT) processing, which induces structural modifications in whey proteins and influences lipid–protein interactions [10]. Butter, on the other hand, is a concentrated source of milk fat, derived from milk or cream, containing some nonfat milk solids and presenting its own crystalline and rheological profile that affects fat network formation in ice cream [11].

Different lipid sources not only modify the viscosity and overrun but also have a profound impact on the formation of the fat crystal network, melt resistance, and mechanical properties of the final product. Dairy fats generally contain higher levels of saturated triacylglycerols and solid fat content (SFC), which favor structured fat aggregation and thermal stability [3]. In contrast, vegetable fats with low-trans content tend to have a higher proportion of unsaturated fatty acids and lower SFC, resulting in a more fluid network that may compromise textural integrity and resistance to melting. Furthermore, UHT-treated cream introduces denatured whey proteins that may reinforce fat–protein interactions and affect overall emulsion stability [1,2,4].

According to the Brazilian Collegiate Board Resolution (RDC) No. 713—dated 1 July 2022 [12]—which regulates ice cream manufacturing in Brazil, the product must meet specific parameters, such as a minimum apparent density of 475 g/L, to be classified as ice cream. This regulation aims to ensure that the final product has the required quality characteristics, including the stability of both lipid and aqueous phases, which are essential for texture, palatability, and structural integrity during consumption.

Although the functional importance of fat in ice cream is well documented, most recent studies have focused on fat reduction or substitution strategies for nutritional optimization. Very few investigations have systematically compared different intact lipid sources—particularly from both dairy and vegetable origins—under controlled formulations [1]. Moreover, the effects of these fats on structural properties before and after aging (maturation) remain largely unexplored in the literature, despite the critical role this step plays in fat crystallization and structural development [2,4].

Therefore, the present study aimed to investigate structural behavior changes in ice cream during the maturation stage of the mixtures and to evaluate the physical properties of the final product as a function of the use of different fat sources: low-trans vegetable fat, butter, UHT cream, and fresh cream. Through rheological analyses and physical testing, this study sought to provide an in-depth understanding of how the selection of the lipid source influences emulsion formation, product stability, and sensory attributes such as texture and melting behavior, thereby contributing to the development of ice creams with superior physical and sensory performance.

## 2. Materials and Methods

### 2.1. Ice Cream Preparation

Four formulations were produced in duplicate with different types of fat: T1—low-trans vegetable fat; T2—80% butter; T3—UHT cream 17%; and T4—fresh cream 25%. The ingredients used and their amounts are shown in Table 1. Whole milk powder (Itambé, Belo Horizonte-MG, Brazil), UHT cream (Itambé, Belo Horizonte-MG, Brazil), fresh cream (Fazenda Bela Vista, Tapiratiba-SP, Brazil), butter (Aviação, São Sebastião do Paraiso-MG, Brazil), and refined sugar União( Camil, São Paulo-SP, Brazil) were purchased in the local market. The powdered additives were kindly provided by Clariant SA (São Paulo-SP, Brazil). Maltodextrin, stabilizer, and emulsifier were supplied by Clariant S.A. (São Paulo-SP, Brazil). Vegetable *trans* fat product PROMULT LT (Bunge, São Paulo-SP, Brazil) was used for transfat formulation. The influence of the maturation stage on the formulation was analyzed, and the formulations are presented in Table 1.

The glucose syrup and sucrose mixtures were diluted with approximately 30% of the total water content. The powdered milk was dissolved in the remaining water under controlled heating, ensuring that the temperature did not exceed 70 °C. Subsequently, the diluted sugar solution was combined with the powdered milk to form the aqueous phase. The fat was heated separately until fully melted, after which the emulsifier was incorporated. The mixture then underwent pasteurization at 75 °C for 15 min in a water bath.

During the process, constant homogenization was maintained using a mechanical shaker at 1200 rpm (Fisaton, Brazil). After homogenization, the mixture was transferred to a 10 L Beaker glassand cooled in an ice bath with continuous stirring until the temperature reached 20 °C. For maturation, the mixture was sealed with plastic film and stored in a refrigerator at 10 °C for 20 h.

After maturation, the mixture was frozen and aerated in a vertical freezer (Polo Sul, model PSV 50, Brazil), with a cooling bath set at −25 °C for approximately 10 min. The freezing process continued until the mass reached −6 °C, achieving the desired texture. The product was then transferred to a plastic container with a lid and frozen for 2 h in a freezer set to −28 °C. Finally, the product was stored in a freezer at −20 °C for further preservation.

#### 2.1.1. Rheology of Mixtures

The rheological properties of the ice cream mixtures were assessed using a Haake Mars II Rheometer (Thermo Electron Corporation, Karlsruhe, Germany) with oscillatory testing, and data analysis was conducted with the RheoWin3 program. Measurements were performed at 4 °C, maintained by a refrigerated bath, using a polished cone–plate sensor (C35/1 Ti). All tests were conducted in triplicate.

To determine the linear viscoelasticity range, stress scans were conducted from 0.01 to 100 Pa. After establishing a fixed stress value within the linear viscoelastic region, frequency sweeps were performed over the range of 0.01 to 10 Hz to generate the mechanical spectrum. The rheological behavior was characterized by evaluating the elastic modulus (G′), the viscous modulus (G″), the ratio between those two moduli (tan δ), and the complex viscosity (η*) as a function of frequency. The mixtures were both analyzed prior to homogenization and cooling (t0) and after 4 h of maturation at 4 °C.

#### 2.1.2. Overrun Determination

Air incorporation into the ice cream matrix, referred to as overrun, is a critical factor influencing the product’s texture, mouthfeel, and apparent density. According to the Brazilian regulation RDC No. 713/2022 [12], edible frozen desserts must present a minimum apparent density of 475 g/L. Considering that the density of the liquid mix (before aeration) typically ranges from 1.0 to 1.1 g/mL, the maximum legally acceptable overrun is approximately 110%, ensuring compliance with the minimum density requirement in the final product.

The overrun was determined by comparing the mass of a fixed volume of the mix before and after the freezing and aeration process. Specifically, a 10 mL aliquot of the liquid mix was weighed in a pre-tared container. The same procedure was conducted with the freshly aerated ice cream. The percentage of overrun was calculated using Equation (1), which was adapted from Clarke (2012) [2] as follows:

(1) $$Overrun\%=\frac{\left[Mix\;weight]-[Ice\;cream\;weight\right]}{\left[Ice\;cream\;weight\right]}\times 100$$

#### 2.1.3. Meltdown Behavior Test

The methodology adapted from [13] was employed to evaluate the relationship between the melting behavior of ice cream and the structural role of the fat phase, particularly the contribution of partial coalescence to network formation. Samples of 100 ± 1 g, previously stored at −20 °C, were placed on a metal grid positioned over a beaker to collect the melted portion. The test was conducted at a controlled ambient temperature of 25 ± 1 °C for 45 min.

The mass of the melted fraction was recorded at 5-minute intervals, and at the end of the experiment, the residual ice cream retained on the grid was quantitatively removed and weighed. From these data, melting curves were constructed for each formulation, enabling comparison of melting rates and the structural integrity imparted by different fat sources and processing conditions.

#### 2.1.4. Colorimetric Analysis

Color measurements of the ice cream samples were performed using a ColorQuest^®^ XE spectrophotometer (HunterLab^®^, Reston, VA, USA), operating under the CIE Lab color space*, with a D65 standard illuminant and a 10° observation angle, in accordance with standardized procedures for food colorimetry. The parameters evaluated were lightness (L*), which indicates brightness on a scale from black (0) to white (100); a*, representing the red–green axis (positive values toward red, negative toward green); and b*, representing the yellow–blue axis (positive values toward yellow, negative toward blue).

Measurements were performed in triplicate directly on the surface of the frozen samples, ensuring homogeneous sampling and minimal influence of surface melting. These data were used to evaluate potential color variations resulting from the different fat sources employed in the formulations.

#### 2.1.5. Texture Analysis

A texture analyzer TA-XT2 (Stable Micro Systems, Surrey, UK) was used to evaluate the mechanical resistance of the ice cream mass immediately after churning at −5 °C. The back extrusion test was selected for its technical suitability in analyzing semi-solid products such as ice cream, providing reliable data on firmness and structural integrity [3]. Each formulation was tested using the AB/E back extrusion probe. Ice cream samples were placed in cylindrical containers, filling approximately two-thirds of the total volume. The probe moved downward at a constant speed of 1 mm/s, as did the pre- and post-test speeds. The probe penetrated 25 mm into the sample, and the maximum force peak required for extrusion was recorded. The results processed using the Texture Expert software version 2.3 (Stable Micro Systems, UK), reflect the internal structure of the product and its resistance to deformation—key attributes for evaluating creaminess, cohesiveness, and spoonability.

The adoption of the back extrusion method is justified by its reproducibility, low sample preparation requirements, and strong correlation with sensory perception of firmness and creaminess in aerated frozen systems. These advantages make it an appropriate and widely used technique in studies involving the rheological and textural properties of ice cream and similar emulsified matrices [14].

#### 2.1.6. Nutrition Information

The nutritional facts for the formulations were based on the Brazilian Table of Food Composition [15], with a reference value of 100 g of product. This procedure was applied to all the formulations evaluated, considering the average values of the nutritional components present in the ingredients used.

#### 2.1.7. Statistical Analysis

Data were subjected to one-way ANOVA followed by Tukey’s test (*p* < 0.05) for comparisons among formulations (T1–T4). Results are expressed as mean ± standard deviation (n = 3). Statistical analyses were performed using MINITAB software 17.3 (Minitab LLC, State College, PA, USA), and all figures were prepared in Microsoft Excel 2019 (Microsoft Corp., Washington, DC, USA).

## 3. Results

### 3.1. Rheology of Mixtures

**Figure 1:**
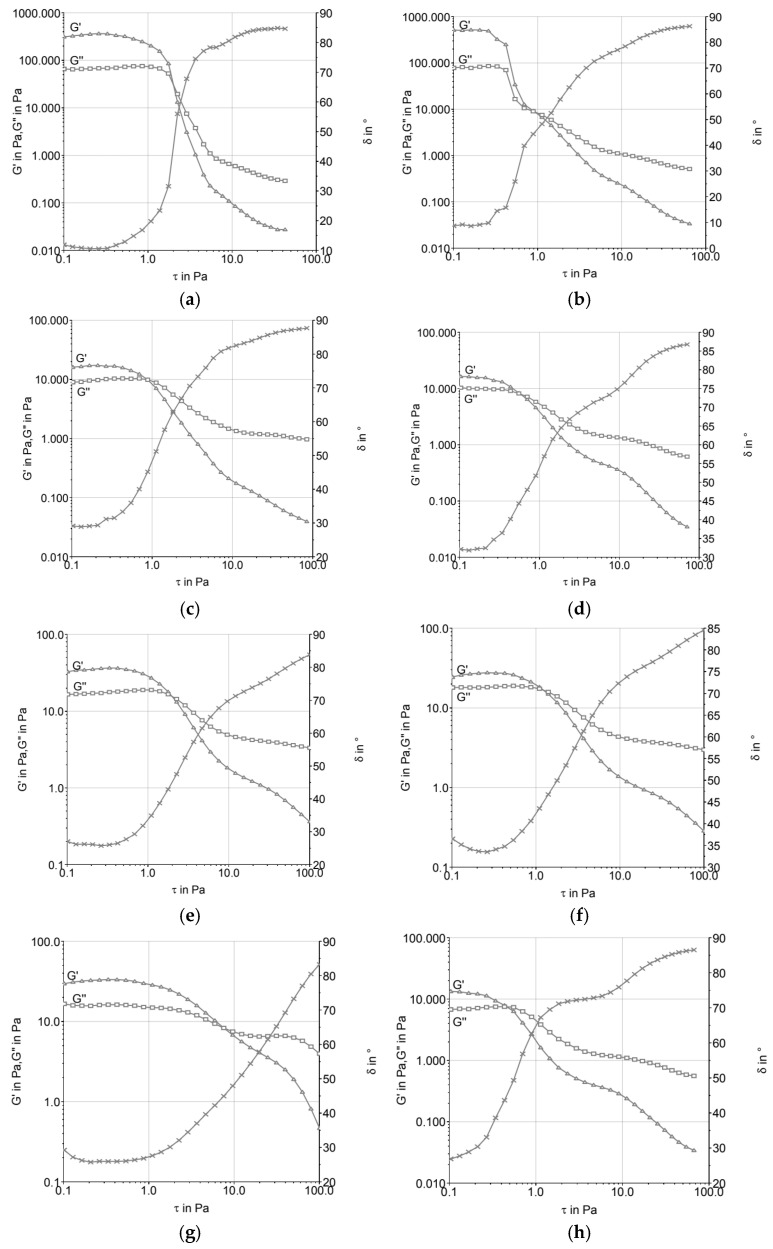
Amplitude sweep of ice cream mixtures before (**a**,**c**,**e**,**g**) and after maturation (**b**,**d**,**f**,**h**): (**a**,**b**) T1 (low-trans vegetable fat); (**c**,**d**) T2 (butter); (**e**,**f**) T3 (UHT cream); (**g**,**h**) T4 (fresh cream).

Figure 1 shows the amplitude sweep curves of the ice cream mixtures before and after maturation. In all treatments, a predominance of elastic behavior (G′ > G″) was observed within the linear viscoelastic region, which indicates the presence of structured networks in the mixes. Differences among the formulations were evident: T2 (butter) presented higher G′ values, while T1 (vegetable fat) showed the lowest. After maturation, T3 (UHT cream) and T4 (fresh cream) exhibited a marked increase in their G′ values, suggesting the reinforcement of interactions during storage.

**Figure 2:**
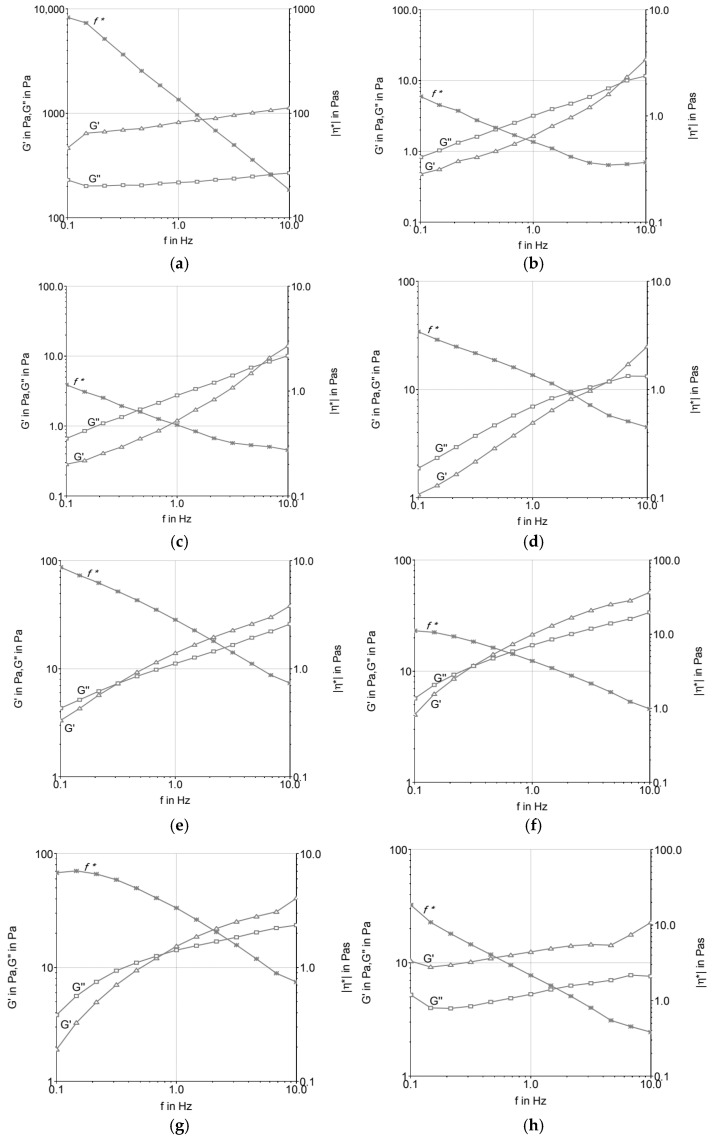
Frequency sweep of ice cream mixtures before (**a**,**c**,**e**,**g**) and after maturation (**b**,**d**,**f**,**h**): (**a**,**b**) T1 (low-trans vegetable fat); (**c**,**d**) T2 (butter); (**e**,**f**) T3 (UHT cream); (**g**,**h**) T4 (fresh cream).

Figure 2 presents the frequency sweep analysis, highlighting the variation in G′ and G″ with oscillatory frequency. T2 maintained elastic dominance across the tested frequencies, while T1 showed frequency dependence, with a crossover between G′ and G″ at lower frequencies. T3 and T4 displayed intermediate curves, with lower absolute values compared with the butter formulation (T2). The results demonstrate that the fat source influenced the viscoelastic profile of the formulations both before and after maturation.

### 3.2. Overrun

**Figure 3:**
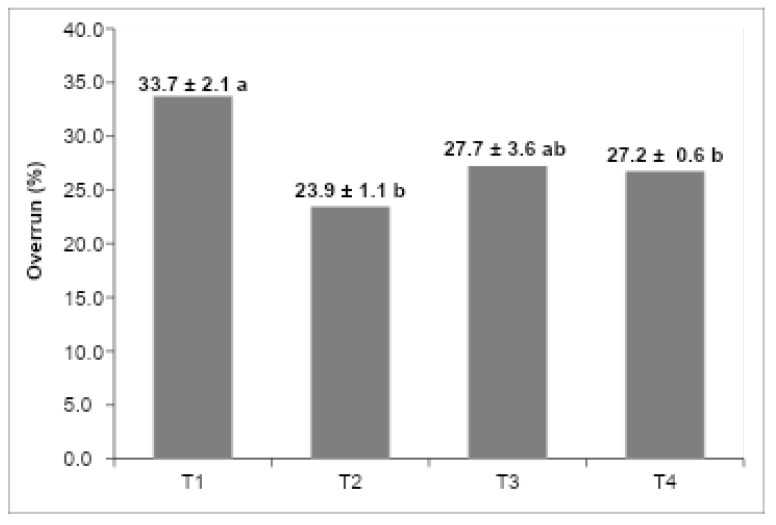
Overrun percentage of the ice cream formulation: (T1) low-*trans* vegetable fat, (T2) butter, (T3) UHT cream, (T4) fresh cream. The values in the columns that do not have the same letters are significantly different at *p* < 0.05 (n:3).

Figure 3 shows the overrun values of the different formulations. T1 (vegetable fat) and T3 (UHT cream) had the highest values, while T2 (butter) showed the lowest. These results reveal that air incorporation varied depending on the fat source used, directly impacting texture and density. Higher overrun values produced lighter structures, while the lower overrun of the butter-based sample indicated a denser and more compact ice cream matrix.

### 3.3. Melting Behavior

**Figure 4:**
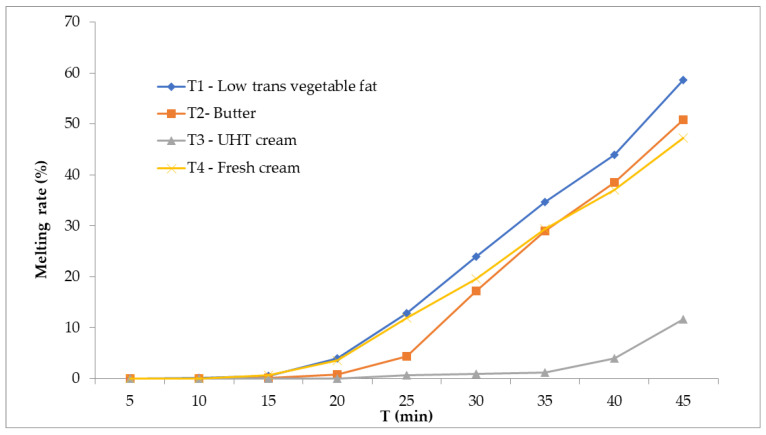
The melting rate of the ice cream formulation: (T1) low-*trans* vegetable fat, (T2) butter, (T3) UHT cream, (T4) fresh cream (n:3).

Figure 4 illustrates the melting behavior of the formulations. The vegetable fat sample (T1) melted more rapidly, while the UHT cream sample (T3) exhibited the lowest weight loss during the test, indicating greater stability against melting. T2 (butter) and T4 (fresh cream) had intermediate values. These differences reflect the distinct interactions between the fat phase, incorporated air, and ice crystals, which collectively influence resistance to structural collapse under thermal stress.

### 3.4. Color

Table 2 presents the instrumental color values (L*, a*, b*) for the samples. All treatments had high L* values, which are consistent with the expected brightness of dairy-based matrices. However, T3 (UHT cream) showed a significantly lower L* (77.51), while T2 (butter) had the highest b* (28.88), indicating a more yellow hue. The a* values were slightly negative in all samples, suggesting a faint greenish tint, with T3 being the lowest (−2.09). These results confirm that the fat source influenced the visual attributes of the ice creams, mainly affecting brightness and yellowness.

### 3.5. Texture

**Figure 5:**
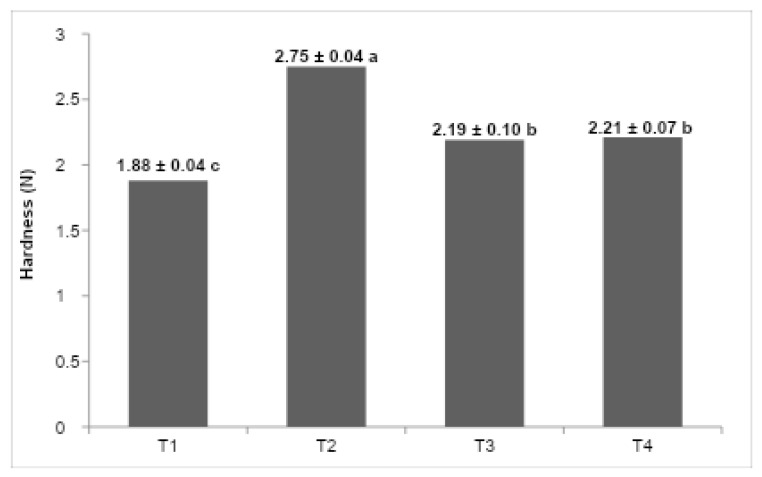
(T1) low-trans vegetable fat, (T2) butter, (T3) UHT cream, (T4) fresh cream. The values in the columns that do not have the same letters are significantly different at *p* < 0.05. Mean ± SD is shown (n:3).

Figure 5 shows the maximum extrusion force values for the formulations. T2 (butter) had the highest firmness, followed by T4 (fresh cream), T3 (UHT cream), and finally T1 (vegetable fat), which presented the lowest value. These differences reflect the influence of lipid composition and crystallization behavior on the mechanical resistance of the ice cream structure.

### 3.6. Nutritional Composition

Table 3 summarizes the nutritional composition of the formulations. The UHT cream sample (T3) showed the highest energy value (126.4 kcal/100 g) due to its higher fat and protein content. Butter (T2) and fresh cream (T4) formulations presented intermediate values, while the vegetable fat formulation (T1) had the lowest caloric content (107 kcal/100 g). Differences were also observed in terms of saturated fat content, with T4 showing the highest amount. These results demonstrate that the fat source significantly affected not only the structural and sensory properties but also the nutritional profile of the final product.

## 4. Discussion

### 4.1. Stress Sweep (Amplitude Sweep)

As shown in Figure 1, the formulations exhibited a crossover of the storage (G′) and loss (G″) moduli around 0.5 Pa, indicating predominantly elastic behavior following the aging process. This response was more pronounced in the samples formulated with vegetable fat (T1) and UHT cream (T3), suggesting a more structured viscoelastic network capable of sustaining deformation under stress. An exception was observed for formulation T2 (butter), which showed a higher crossover point near 1.0 Pa, indicating reduced elastic dominance at lower stress levels and potential structural fragility. These findings are consistent with Brazmi et al. [16], who demonstrated that variations in lipid composition can significantly affect the rheological profile of frozen systems, with direct implications for stability and textural performance.

Formulations T3 (UHT cream) and T4 (fresh cream) displayed lower values for both G′ and G″, which may be attributed to their compositional characteristics, particularly the higher proportion of unsaturated lipids and reduced solid fat content (SFC). These factors contribute to less organized fat crystal networks, resulting in decreased resistance to deformation and reduced mechanical integrity. In the case of UHT cream, thermal processing may promote partial denaturation of whey proteins and alter fat–protein interfacial interactions, potentially impairing the development of a cohesive microstructure. The greater fluidity of these formulations at near-zero temperatures likely undermines emulsion stability upon partial melting, contributing to more flow-prone behavior under stress conditions.

In the case of UHT cream, the ultra-high-temperature sterilization process can cause changes in protein structures and fat globules, leading to a loss of stability at higher temperatures. This phenomenon, when observed in the rheological properties of ice cream, can result in a more fluid product that does not resist mechanical stress, such as during processing and freezing [1]. This behavior was discussed by [17], who pointed out that stabilizers present in ice cream emulsions play a crucial role in modulating rheological characteristics, controlling the viscosity and texture of the final product.

Ice water plays a fundamental role in the rheological behavior and microstructure of ice creams. The presence of solid water, i.e., ice, dictates the rigidity of the sample, which is expressed in the elastic modulus (G′). Ice creams with higher amounts of ice water tend to exhibit greater hardness and rigidity, which are crucial for ensuring proper handling during consumption and for maintaining stability over time. The formation of ice during freezing and its interaction with the lipid and protein phases of the ice cream matrix also affect the product’s microstructure, as noted by [18], who discussed the importance of this solid fraction in the product’s behavior.

The effect of ice water can also be linked to the rheological behavior of ice cream emulsions. The formation of ice and its interaction with lipid and protein phases influences not only viscosity but also texture properties. The hardness of ice cream is a critical parameter, particularly in the context of sales and consumption, where texture can be a key determinant of product acceptance. Studies by [19] suggest that modulating the amount of ice water in the system is crucial for controlling the sensory characteristics of ice cream, such as creaminess and optimal melt performance.

The crystallization of triacylglycerols (TAGs), the primary components of fats in ice cream, is a crucial phenomenon for determining the product’s structural and textural properties. The crystallization behavior of TAGs is greatly influenced by factors such as fat composition (saturated vs. unsaturated) and melting temperature. As noted by [20], trans fatty acids crystallize faster than their cis isomer counterparts, which can lead to a better structuring and formation of a more stable lipid network in products like ice cream.

The presence of unsaturated fats can modify the crystallization rate of TAGs, as discussed by [21]. The formation of smaller fat globules, which occurs with a higher content of unsaturated fatty acids, tends to result in more stable and smoother emulsions, which are desirable for ice cream products. However, controlling the crystallization rate is essential, as too-rapid crystallization can lead to the formation of large ice crystals, resulting in a grainy texture.

After the maturation of ice cream emulsions at low temperatures, the physical and rheological properties change due to the hydration of stabilizers. As discussed by [22], the hydration of stabilizers like guar gum, xanthan gum, or gelatin increases the viscosity of emulsions, promoting improved stability and texture during storage. The presence of emulsifiers and stabilizers, such as glycerol esters and milk proteins, can significantly influence flow and resistance properties, leading to a dynamic balance between the elastic and viscous moduli [18,22].

The different rheological behaviors observed between formulations with butter, fresh cream, and UHT cream reflect not only differences in physical properties of these ingredients but also in crystalline structures and melting/solidification processes [18]. Saturated fats, like butter, offer greater control over texture and rheological stability, while unsaturated and processed fats, such as those in UHT cream, have a more fluid behavior, with less stability under certain conditions. Differences in crystallization properties and the stability of emulsifiers result in significant variations in final texture and behavior during the handling and storage of ice cream [17,22].

### 4.2. Frequency Sweep

The variation in material properties with respect to strain and stress rates was investigated using an oscillatory frequency sweep test. This test demonstrates how the elastic and viscous behavior of a material oscillates, and it is possible to analyze the complex viscosity as a function of frequency, describing the total resistance to dynamic shear. In this test, the complex viscosity either increases or decreases while maintaining constant tension [23]. Based on the parameters tested, all formulations indicated that frequency could be proportionally increased with the increase in the elastic and viscous moduli, as well as the decrease in the complex viscosity curve. This suggests the subordination of the moduli to frequency, thus characterizing the viscoelastic fluid behavior, where the molecules tend to orient themselves in the direction of the applied force. Under shear stress, the material typically behaves as a pseudoplastic fluid, i.e., a non-Newtonian fluid where the viscosity decreases with increasing shear rate [23]. This behavior was observed in most samples, as reported by [20,22,24] for ice cream systems.

Among the formulations tested (Figure 2), those produced with fresh cream and UHT cream presented the lowest frequency resistance, being below 1 Hz. Additionally, these formulations showed the lowest loss modulus (G″) and storage modulus (G′), indicating a less rigid structure compared to other formulations. This suggests that the fat composition and the manufacturing process (such as the UHT treatment of UHT cream) significantly influence the rheological properties of the system. The lower G′ and G″ values of these formulations indicate a weaker gel structure, with less resistance to deformation under applied stress.

These findings are consistent with the observations made by [16], who emphasized the structural sensitivity of ice cream mixtures based on dairy fat during the maturation phase at 4 °C. At this stage, emulsions undergo structural changes due to the reorganization of fat globules and the hydration of stabilizers, which affects the elastic and viscous properties of the ice cream base. The UHT cream, for instance, can undergo significant changes in its fat globule membrane during processing, leading to a destabilized fat phase, which exhibits more fluid-like behavior under oscillatory shear tests.

It is important to note that the maturation process plays a crucial role in altering the rheological properties of ice cream formulations, as pointed out by [19,22]. During maturation, emulsifiers and stabilizers hydrate, which increases viscosity and alters the gel network. As a result, formulations with low-trans-fat content exhibit higher G′ values and a more elastic behavior, while formulations with fresh cream and UHT cream, due to their weaker lipid structures and lower G′ values, may not exhibit the same level of rigidity after maturation, resulting in higher tan δ values and a behavior more dominated by viscosity. This behavior is consistent with [16], who noted that emulsions made with dairy fat exhibit a prevalence of the elastic modulus after maturation at low temperatures.

The shear-thinning behavior observed in the formulations, especially those containing fresh cream and UHT cream, suggests that these systems are well suited for processing under high shear conditions, which are typical of ice cream production. The results from the oscillatory shear test reflect the ability of these emulsions to flow more easily under high shear stress, contributing to a smoother, creamier texture when whipped. However, the lower G′ values observed in these formulations might indicate a softer texture in the final product, which could affect the mouthfeel and structural integrity of the ice cream.

In contrast, samples with higher fat content or with more stable fat systems demonstrated elevated G′, G″, and complex viscosity values. This behavior indicates enhanced fat crystal packing and greater contribution of partial coalescence to the viscoelastic network, conferring superior firmness and stability. The balance between elasticity (G′) and viscosity (G″) is critical for maintaining the microstructural integrity of ice cream throughout processing, storage, and consumption, ultimately ensuring consistent texture and consumer acceptability.

There was a significant reduction in complex viscosity at high frequencies, which caused the disaggregation of fat particles in the system. It was clear that the viscosity of the mixtures depends on the type of fat and hydrocolloids added to the formula and the size of these particles in the matrix. It was possible to verify that the ice creams produced with low-*trans* fat (T1) and butter (T2) were the most rigid products and had the most ice crystals among the formulations tested. Therefore, we highlight the thesis that low-*trans* vegetable fat suffers less membrane destabilization because it has a large amount of palmitic acid (16:0), ranging from 35 to 47%. This high level of saturated fatty acids is disadvantageous for the destabilization of fat globules, as this destabilization will be greater the more unsaturated and longer the chains of vegetable fat used in the formulation. Furthermore, mono- and diacylglycerol emulsifiers can interact better through the hydrophobic region of the molecules [24].

However, formulations T2 and T3, produced with milk fat, demonstrated similar and lower values (<1) of tan δ, thus indicating the existence of a remaining microstructure. This can be attributed in part to the presence of proteins in association with stabilizers. Furthermore, the fact that these formulations achieved similar G′ values above 0.5 Hz frequency suggested that interactions between milk proteins and fat occurred to ensure a structured product. The tan δ values were below 1 at high frequencies, G′ was greater than G″, proposing that the molecules have less mobility, and the product has solid and not liquid properties. The ice cream prepared with milk fat (T2, T3, and T4) presented a lower slope of the curves and differentiated profiles, indicating greater stability.

### 4.3. Overrun Determination

The calculated overrun values for the evaluated ice cream formulations are depicted in Figure 3. Overrun, defined as the volume increase due to air incorporation during freezing and whipping, is a paramount quality parameter influencing the texture, mouthfeel, and overall consumer acceptance of frozen desserts [25,26]. The extent of air incorporation is inherently dependent on the interplay between the fat globule network, milk proteins, emulsifiers, stabilizers, and ice crystal morphology, all of which modulate the viscoelastic properties and stability of the continuous phase [5,27].

Among the tested formulations, T3 (UHT cream) and T1 (low-trans vegetable fat) exhibited the highest overrun values, indicating superior air entrapment and retention capacities. The elevated overrun in T3 is attributable not only to the relatively stable crystalline structure of the UHT cream fat fraction but also to the intrinsic presence of hydrocolloidal stabilizers incorporated during industrial processing [28,29]. These stabilizers synergistically interact with emulsifiers and anticrystallization agents added in the ice cream mix, enhancing the interfacial stability of fat globules and improving the viscosity of the unfrozen serum phase, which collectively promote the formation and maintenance of a cohesive partial coalescence fat network [18,19].

While total fat content remains a significant factor in determining overrun, controlled destabilization of the fat membrane during mechanical agitation is critical for partial coalescence, which stabilizes air cells within the matrix and confers elasticity to the continuous phase [20,30]. In contrast, formulation T2 (butter) presented the lowest overrun, a phenomenon consistent with its comparatively weaker fat network, as corroborated by lower values of the storage (G′) and loss moduli (G″) in rheological frequency sweep tests (Figure 2). These rheological findings indicate diminished structural strength and elastic behavior, which compromise the product’s ability to trap and stabilize air bubbles, thus limiting overrun [12,21].

Moreover, the rheological profiles reveal a positive correlation between higher elastic modulus and increased overrun, emphasizing the role of fat–protein interactions and stabilizer systems in dictating air incorporation efficiency [1,22]. Stabilizers present in both the UHT cream and the ice cream base reduce excessive ice crystal growth by inhibiting water mobility and recrystallization, leading to a smoother texture and further aiding the stabilization of the aerated structure [14,23].

Consequently, the superior overrun observed in T3 reflects the technological advantages conferred by the integration of lipid stabilizers and complex emulsifier systems, which collectively optimize the viscoelastic properties of the ice cream matrix. This formulation strategy enhances the retention of incorporated air, improves creaminess and sensory perception, and contributes to extended shelf-life through increased storage stability [17,18]. Thus, the use of UHT cream enriched with stabilizers, in combination with a tailored rheological formulation, represents an effective approach for the production of premium ice cream products with enhanced structural and sensory qualities.

### 4.4. Melting Behavior

The melting behavior of ice cream constitutes a pivotal quality attribute, intimately linked to both its sensory perception and physicochemical stability, as it directly reflects the microstructural integrity of the frozen matrix and the interactions among ice crystals, fat networks, and serum phases [16,18]. Figure 4 presents the cumulative melting profiles of the four formulations over a 45-min interval under rigorously controlled ambient conditions, with the initial sample masses standardized to 100 g to enable valid comparative analyses of absolute melt loss and temporal melting kinetics [1].

Formulation T1, incorporating low-trans vegetable fat, exhibited the highest melting rate, losing approximately 63 g of mass (63%) over the test duration. This pronounced melt susceptibility can be ascribed to the intrinsic physicochemical characteristics of industrially modified vegetable fats, which generally display a fractionated triglyceride composition and possess less stable polymorphic crystalline structures compared to dairy fats [31]. Consequently, these fats exhibit diminished capacity for partial coalescence and the formation of an interconnected fat globule network during the freezing process, thereby compromising the mechanical rigidity and melt resistance of the ice cream matrix [18,20]. The limited formation of interglobular bridges results in a weaker three-dimensional fat network that inadequately immobilizes unfrozen water, accelerating melting kinetics [31,32].

Conversely, formulation T3, based on UHT cream, demonstrated the most robust melting resistance, with only about 13 g (13%) of mass loss, indicating a highly cohesive and stable frozen structure. This enhanced performance is likely mediated by the denaturation of whey proteins induced during the ultra-high-temperature treatment, which facilitates the formation of protein–fat aggregates [15,31]. These aggregates contribute to the reinforcement of the fat–protein matrix, effectively entrapping free water within the microstructure and mitigating phase separation during melting [14,17,31]. Additionally, the UHT process may alter emulsification dynamics, enhancing fat globule interface stability and favoring a more resilient network formation upon freezing [19,25].

The intermediate melting profiles observed for T2 (butter; ~50% melt) and T4 (fresh cream; ~46% melt) reflect their compositional and processing attributes. Butter, rich in short- and medium-chain saturated triglycerides, can form rigid crystalline networks; however, its structural contribution is highly dependent on emulsification efficiency and crystallization kinetics, which are factors that govern the extent of fat partial coalescence and network continuity [18]. Fresh cream, while retaining native emulsifying milk proteins such as caseins, undergoes minimal thermal treatment, which may limit protein denaturation and subsequent aggregate formation necessary for robust fat–protein network stabilization [20,22]. Moreover, variations in total solids and serum viscosity between fresh and UHT cream further influence matrix rheology and melting resistance [23,33].

Another relevant factor contributing to the superior melt resistance of T3 is its lower overrun, resulting in a denser microstructure with reduced air cell volume. The diminished air incorporation increases the thermal mass and reduces the specific surface area exposed to ambient temperature, thereby retarding heat transfer and melting rates [14,22]. This attribute is especially desirable in premium or regionally targeted ice cream products intended for warm climates, where prolonged shape retention and slow melting are critical for consumer satisfaction and product differentiation [27,34].

In summary, the melting profiles elucidate the fundamental role of fat source composition, thermal processing, and microstructural organization in determining the melt stability of ice cream. The findings affirm that the structural functionality of protein–fat networks, modulated by ingredient characteristics and processing parameters, is paramount for controlling melting behavior, thereby influencing sensory attributes and shelf-life performance [14,35].

### 4.5. Color Determination

Color is a critical sensory attribute in the acceptance of ice cream, as it directly shapes consumer expectations regarding flavor, freshness, and overall product quality [14,36]. Objective measurements were obtained through reflectance colorimetry using the CIE Lab* color space, which quantifies the three-dimensional coordinates L* (lightness), a* (red–green axis), and b* (yellow–blue axis). This approach provides reproducible and comparable parameters for visual characterization and is widely employed in dairy-based colloidal systems due to its ability to capture subtle compositional and processing-induced variations [5,37,38,39].

The L* values were consistently high across all formulations, reflecting the typical optical behavior of dairy colloids in which light scattering is mainly governed by fat globule dispersion and casein micelle distribution within the aqueous matrix [5,37]. However, treatment T3 (UHT cream) exhibited a significantly reduced L* value (77.51) compared with T1 (81.01), T2 (80.25), and T4 (79.36). This darker appearance may be associated with the formation of early Maillard reaction products and non-enzymatic browning intermediates generated during severe heat treatment [2,27]. Compounds such as hydroxymethylfurfural (HMF) and furosine are known to absorb visible light, thereby lowering diffuse reflectance [40,41,42]. Since no such markers were quantified in this study, the explanation remains hypothetical and requires confirmation by targeted chemical analyses.

The a* coordinate was slightly negative in all samples, indicating a faint greenish hue typical of dairy products and which is often attributed to trace chlorophyll derivatives or subtle scattering effects [39,43]. T3 showed the most negative a* value (−2.09), which could be linked to microstructural modifications from UHT processing that alter light absorption and scattering [41]. Nonetheless, the small absolute magnitude of these values suggests that such differences are unlikely to be perceptible without complementary visual or spectrophotometric evaluation.

All samples exhibited positive b* values consistent with the characteristic yellowish coloration of fat-rich dairy matrices [41,43]. The butter-based formulation (T2) showed the highest b* (28.88), attributable to the presence of β-carotene and other lipophilic pigments concentrated in milk fat [41]. The greater yellowness in T2 may also be accentuated by reduced emulsification efficiency, leading to larger fat globule clusters that intensify light absorption [14,19]. Conversely, T3 displayed a reduced b* (25.74), plausibly due to carotenoid degradation during sterilization, in line with documented thermal sensitivity of these pigments [40,41].

It should be emphasized that this study did not include pigment-specific analyses such as high-performance liquid chromatography (HPLC) or UV–Vis spectrophotometry. As a result, the interpretation of L* reduction and b* shifts as outcomes of Maillard intermediates or carotenoid degradation should be considered with caution. Future investigations combining instrumental colorimetry with molecular-level characterization and sensory validation are warranted [22,36].

In summary, the colorimetric data demonstrate that fat source exerts a decisive influence on the visual attributes of ice cream. Variations in L* and b* values reflect intrinsic differences in lipid composition, pigment content, and thermal processing history of the fat ingredients. These findings underscore the strategic role of fat selection in shaping the final visual appeal of ice cream and, consequently, its acceptance in the marketplace [14,41].

### 4.6. Texture Determination

The texture of ice cream is a pivotal quality attribute that profoundly influences consumer perception and overall product acceptability. This attribute emerges from the complex interplay among ice crystals, fat globules, air cells, and the frozen serum matrix, which collectively determines the microstructure and mechanical integrity of the product [4,14]. Instrumental texture analysis provides objective quantification of critical mechanical parameters such as firmness, cohesiveness, and resistance to deformation, which closely correlate with sensory attributes including creaminess, hardness, and melt profile [3,22]. Understanding and controlling these parameters is essential for formulating ice creams that meet both technological requirements and consumer expectations.

In this study, the extrusion test was selected to evaluate ice cream firmness or hardness due to its superior sensitivity in mimicking the consumer’s scooping action compared to the conventional cone penetration test [4,19]. Furthermore, extrusion testing engages a larger sample volume, enhancing representativeness in heterogeneous semi-solid systems such as ice cream, wherein the tri-dimensional structure results from the interaction between air cells, fat crystal networks, and ice crystals [22,27]. This methodological advantage allows for a more comprehensive assessment of structural nuances that critically influence rheological and sensory outcomes.

Statistical analysis revealed significant differences (*p* < 0.05) in firmness across formulations (Figure 5). The butter-based formulation (T2) exhibited the highest maximum extrusion force, indicative of a more rigid and mechanically robust matrix, followed sequentially by fresh cream (T4), UHT cream (T3), and vegetable fat (T1), which presented the lowest firmness values. These differences can be attributed to the distinct lipid composition and crystallization behaviors inherent to each fat source [14,25]. Butter, characterized by a high solid fat content at freezing temperatures (~−18 °C) and polymorphic fat crystallization, forms a stable crystalline network that restricts water mobility and enhances mechanical strength [4], thus reinforcing the ice cream’s structural integrity.

Additionally, the butter formulation demonstrated the lowest overrun, which correlates with increased density and fewer air-induced structural discontinuities, thereby elevating resistance to deformation [22,27]. Conversely, the vegetable fat-based formulation, although plastically stable, possesses a reduced solid fat fraction at freezer temperatures and contains emulsifiers that impede partial coalescence of fat globules, limiting the formation of aggregated fat structures essential for mechanical reinforcement [14,25]. Consequently, this formulation yields a softer texture with higher deformability under compressive forces.

Intermediate firmness values observed for the UHT cream (T3) and fresh cream (T4) formulations likely reflect differences induced by thermal processing. The reduced firmness of the UHT cream may be associated with protein denaturation caused by high-temperature treatment, which can compromise fat emulsification and water-holding capacity, resulting in lower matrix stability and mechanical strength [19,29]. However, no direct measurements of protein denaturation (such as solubility assays or protein fraction analyses) were performed in this study; thus, this explanation should be considered a plausible mechanism supported by literature rather than a confirmed finding. Fresh cream, by retaining native milk proteins, favors greater stabilization of the continuous phase and facilitates the formation of a more cohesive fat network, resulting in slightly higher firmness despite its lower fat content compared with butter [32].

The results presented here exclusively reflect the measurement of hardness through the extrusion test. Other relevant texture parameters—such as cohesiveness, elasticity, and adhesiveness—were not determined. This means that the data describes the resistance to compression with precision but do not capture the multi-dimensional nature of ice cream texture. Future investigations should incorporate a broader instrumental texture profile, combined with sensory analyses, to provide a more comprehensive understanding of the relationship between structure, mechanical properties, and consumer-perceived quality [44,45].

### 4.7. Nutritional Composition

The comprehensive nutritional profiling of the ice cream formulations (T1, T2, T3, and T4), detailed in Table 3, underscores significant variability in macronutrient distribution and lipid composition. These parameters are paramount in both product development and nutritional risk assessment [19,26,45]. The precise quantification of energy content, protein, carbohydrates, and total and saturated fats, alongside sodium levels, enables a multi-dimensional evaluation of the formulations’ nutritional quality and their alignment with dietary recommendations and public health policies [46,47].

The energy values were distinctly heterogeneous, with formulation T2 demonstrating the lowest caloric density (117.5 kcal/100 g), contrasted by T3′s highest caloric load (126.4 kcal/100 g). This variance reflects the differential contributions of lipid and protein fractions, which are the predominant macronutrients influencing energy density in frozen dairy matrices [7,18]. The carbohydrate content across samples remained generally consistent, save for T3, which exhibited a relatively elevated carbohydrate concentration (23.3 g/100 g). This anomaly is plausibly linked to the inclusion of polysaccharide-based stabilizers or other carbohydrate-rich additives, which are commonly utilized in UHT cream formulations to modulate rheology and shelf-life [44,48].

Protein quantification further delineated the formulations, with T3 displaying a notable increase (4.8 g/100 g) relative to the other samples (3.2–3.3 g/100 g), corroborating the elevated protein content characteristic of UHT-treated dairy creams vis-à-vis butter or plant-based fats [18,49]. The nutritional implications of heightened protein levels extend beyond caloric contribution, potentially enhancing satiety responses and supporting anabolic processes, making them particularly relevant for demographic groups with elevated protein requirements such as the elderly, athletes, or clinical populations [50,51]. A pronounced divergence was observed in saturated fat content, which is a critical determinant in cardiovascular risk modulation [47,52]. The low-trans vegetable-fat-based formulation (T1) exhibited the minimal saturated fat fraction (5.2 g/100 g), being consistent with lipid profiles dominated by unsaturated fatty acids prevalent in plant-derived fats [50,52]. Conversely, T4 (fresh cream) manifested the highest saturated fat concentration (27.5 g/100 g), reflective of the intrinsic fatty acid composition of full-fat dairy lipids, which include significant proportions of palmitic and myristic acids that are both implicated in lipid metabolism and cardiovascular health outcomes [1,53]. The intermediate saturated fat levels in T2 (butter) and T3 (UHT cream) (7.3 and 8.3 g/100 g, respectively) reflect compositional nuances influenced by moisture content, processing modalities, and fat crystallization behavior [28].

The sodium concentration also varied significantly, with T3 presenting the highest level (77.6 mg/100 g), which is attributable to the incorporation of salt-containing stabilizers and preservatives often added to UHT cream formulations to ensure microbiological stability and texture retention [53]. In contrast, T4′s sodium content (49.7 mg/100 g) was the lowest, aligning with its composition of minimally processed dairy devoid of additive inclusion [1,22]. Given the global emphasis on sodium reduction strategies to mitigate hypertension and cardiovascular disease risk [46,47], these findings emphasize the criticality of ingredient and process selection in product formulation.

The utilization of advanced nutritional software tools facilitated precise compositional analysis and enabled iterative formulation adjustments to meet stringent regulatory and health-oriented targets [19,54]. This methodological integration exemplifies the intersection of food science and nutritional epidemiology, addressing the contemporary challenge of balancing sensory attributes, product functionality, and health promotion [14,51]. Collectively, the comparative nutritional insights derived from these formulations substantiate the pivotal role of fat source selection in optimizing ice cream formulations for targeted consumer groups, aligning product innovation with evolving nutritional paradigms and market demands [39,55].

### 4.8. Correlation Analysis Among Indicators

The integration of physicochemical, rheological, and textural parameters allowed for the identification of significant correlations among the evaluated variables. A strong negative correlation was observed between overrun and hardness (r = −0.87; *p* < 0.05), indicating that formulations with higher air incorporation, such as T1 (31.3%) and T3 (29.7%), exhibited lighter and less resistant matrices, while T2, with lower overrun (18.5%), resulted in the highest structural firmness [3,12]. This behavior reinforces the role of aeration as a determinant of texture, since the presence of air cells reduces density and increases softness [1,2].

In addition, a positive correlation was verified between storage modulus (G′) after maturation and melting resistance (r = 0.81; *p* < 0.05). The T3 formulation, produced with UHT cream, presented the highest G′ (>0.5 Hz) and simultaneously the lowest mass loss during the melting test (13 g in 45 min), confirming that more elastic systems provide greater thermal resistance and structural stability [16,20]. The protein denaturation effect induced by UHT treatment seems to have contributed to the coupling between whey proteins and fat globules, favoring the formation of a cohesive and stable network [10,19]. Furthermore, tan δ values greater than 1, observed in T1, were associated with a predominance of viscous behavior and with faster melting (63 g in 45 min), suggesting reduced ability to retain unfrozen water and lower continuity of the fat–protein network [22,24]. This finding is consistent with the hypothesis that vegetable fats with lower solid fat content at −18 °C and less stable polymorphism reduce the ability to form robust three-dimensional structures [11,28].

The correlations also indicate a functional coupling between texture and thermal stability: The T2 formulation, with the highest hardness (extrusion force), showed the lowest overrun and intermediate melting (~50 g), suggesting that the rigid crystalline network formed by butter increases mechanical resistance but does not guarantee maximum stability against heating [7,21]. This result highlights the need to consider the interaction among lipid crystallinity, partial coalescence, and system elasticity as determinants of overall ice cream performance [18,26].

Therefore, the correlation analysis reveals consistent structural relationships: (i) higher overrun → lower hardness; (ii) higher G′ → greater melting resistance; (iii) tan δ > 1 → higher susceptibility to melting. These findings support a clear causal logic of the type “fat source → structure → functional quality,” as suggested by both classical and recent studies on structural stabilization in ice cream systems [1,20,27,53].

## 5. Conclusions

This study clearly demonstrated that the choice of fat source plays a crucial role in shaping the structural and physical properties of ice cream during maturation and in the final product. The different fats tested—ranging from low-trans vegetable fat to traditional dairy fats like butter and creams—uniquely influence emulsion stability, texture, and melt resistance. Ice creams formulated with low-trans vegetable fat stood out for their lighter texture and greater air incorporation, resulting in a softer, more pleasant mouthfeel. In contrast, formulations using butter and dairy creams exhibited greater firmness and melting resistance, which are qualities highly valued for a classic ice cream sensory experience. These findings deepen our understanding of the complex interactions between fat type and ice cream structure, paving the way for innovative formulations that combine high quality, superior physical performance, and consumer satisfaction. Ultimately, this work advances ice cream technology by offering strategic formulation options tailored to diverse market demands and sensory preferences.

## Figures and Tables

**Table 1:** Ice cream formulations.

Ingredients (%)	T1	T2	T3	T4
Water	59.8	58.4	16.4	43.3
Sucrose	12.0	12.0	12.0	12.0
Glucose syrup	6.0	6.0	6.0	6.0
Whole milk powder	12.0	12.0	12.0	12.0
Stabilizers and Emulsifiers	1.3	1.3	1.3	1.3
Low-*trans* vegetable fat	8.9	-	-	-
Butter 80%	-	10.3	-	-
UHT cream 17%	-	-	52.3	-
Fresh cream 25%	-	-	-	25.4

Note: All formulations were balanced with the same variation of fat (12.0%), nonfat milk solids (8.5%), sugar (18.0%), and total solids (39.8%). The formulations were prepared one day before the analysis and reproduced as many times as necessary to obtain the total amount required (batch of 3 kg).

**Table 2:** Color analysis—ice creams: (T1) low-trans vegetable fat, (T2) butter, (T3) UHT cream, (T4) fresh cream.

Treatments	T1	T2	T3	T4
L*	81.01 ± 0.32 a	80.25 ± 0.81 a	77.51 ± 0.41 b	79.36 ± 0.24 a
a*	−1.89 ± 0.02 a	−1.88 ± 0.11 b	−2.09 ± 0.30 c	−1.94 ± 0.04 a
b*	23.26 ± 0.13 a	28.88 ± 0.41 b	25.74 ± 0.60 c	24.34 ± 0.11 a

The values in the lines that do not have the same letters are significantly different at *p* < 0.05 (n:3).

**Table 3:** Nutritional information: (T1) low-trans vegetable fat, (T2) butter, (T3) UHT cream, (T4) fresh cream.

Nutritional Information				
Serving Size 100 g				
	T1	T2	T3	T4
Energy value	107 kcal	117.5 kcal	126.4 kcal	122 kcal
Carbohydrates	21.2 g	21.2 g	23.3 g	21.7 g
Proteins	3.3 g	3.3 g	4.8 g	3.2 g
Total fat	10.4 g	11.6 g	11.9 g	12.1 g
Saturated fat	4.4 g	7.3 g	8.3 g	27.5 g
Trans fat	0.0 g	0.0 g	0.0 g	0.0 g
Dietary fiber	0.0 g	0.0 g	0.0 g	0.0 g
Sodium	52.2 mg	50.8 mg	77.6 mg	49.7 mg

## Data Availability

The data presented in this article will be openly available from the author upon request.
